# Missense variants in KATNA1 alter microtubule dynamics and underlie dominant macular dystrophy

**DOI:** 10.21203/rs.3.rs-8880283/v1

**Published:** 2026-07-07

**Authors:** Karolina Kaminska, Abigail R. Moye, Mathieu Quinodoz, Elena A. Zehr, Amel Aiteur, Giacomo Calzetti, Pilar Barberán-Martínez, Laura Kuehlewein, Jan-Philipp Bodenbender, Miriam Ehrenberg, Samir Khandhadia, Dinah Zur, Shiri Zayit-Soudry, Valeria Barili, Enrico Ambrosini, Giorgia Ottonelli, Anselmo Feliciano-Sánchez, Isabelle Müller, Sandrine Wallerich, Patricia Galliker, Alexandre P. Moulin, Theresia Zuleger, Tobias B. Haack, Bernd Wissinger, Katarina Stingl, Gema García-García, Moreno Menghini, Ajoy Vincent, Elise Héon, Omar A. Mahroo, Andrew J. Lotery, Andrew R. Webster, Gavin Arno, José M. Millán, Tamar Ben-Yosef, Michel Michaelides, Siying Lin, Susanne Kohl, Antonina Roll-Mecak, H. Viet Tran, Carlo Rivolta

**Affiliations:** 1 Institute of Molecular and Clinical Ophthalmology Basel (IOB), Basel, Switzerland; 2 Department of Ophthalmology, University of Basel, Basel, Switzerland; 3 Division of Genetics and Genome Biology, School of Biological and Biomedical Sciences, University of Leicester, Leicester, UK; 4 Cell Biology and Biophysics Unit, National Institute of Neurological Disorders and Stroke, Bethesda, MD, USA; 5 Jules-Gonin Eye Hospital, Fondation Asile des Aveugles, University of Lausanne, Lausanne, Switzerland; 6 Vista Vision Eye Clinic, Brescia, Italy; 7 Department of Medicine and Surgery, University of Parma, Parma, Italy; 8 Molecular, Cellular, and Genomic Biomedicine Group, Health Research Institute La Fe, Valencia, Spain; 9 Joint Unit CIPF-IIS La Fe Molecular, Cellular and Genomic Biomedicine, Valencia, Spain; 10 Center for Ophthalmology, University Eye Hospital, University of Tübingen, Tübingen, Germany; 11 Department of Ophthalmology, Schneider Children's Medical Center of Israel, Petach Tikva, Israel; 12 Gray Faculty of Medical and Health Sciences, Tel Aviv University, Tel Aviv, Israel; 13 Southampton Eye Unit, University Hospital Southampton, Southampton, UK; 14 Division of Ophthalmology, Tel Aviv Sourasky Medical Center, Tel Aviv, Israel; 15 Department of Ophthalmology, Rabin Medical Center, Petach Tikva, Israel; 16 Medical Genetics, University Hospital of Parma, Parma, Italy; 17 Department of Ophthalmology, La Fe University and Polytechnic Hospital, Valencia, Spain; 18 Eye Clinic, Luzerner Kantonsspital (LUKS), Luzern, Switzerland; 19 Institute of Medical Genetics and Applied Genomics, University of Tübingen, Tübingen, Germany; 20 Centre for Rare Diseases, University of Tübingen, Tübingen, Germany; 21 Institute for Ophthalmic Research, Center for Ophthalmology, University of Tübingen, Tübingen, Germany; 22 Center for Biomedical Network Research on Rare Diseases (CIBERER), Instituto de Salud Carlos III, Madrid, Spain; 23 Ophthalmology Service, Institute of Clinical Neurosciences of Southern Switzerland (INSI), Ente Ospedaliero Cantonale (EOC), Lugano, Switzerland; 24 Faculty of Biomedical Sciences, University of Southern Switzerland (USI), Lugano, Switzerland; 25 Department of Ophthalmology and Vision Sciences, The Hospital for Sick Children and University of Toronto, Toronto, Ontario, Canada; 26 NIHR Biomedical Research Centre, Moorfields Eye Hospital and the UCL Institute of Ophthalmology, London, UK; 27 UCL Institute of Ophthalmology, University College London, London, UK; 28 Department of Ophthalmology, St Thomas’ Hospital, London, UK; 29 Faculty of Medicine, University of Southampton, Southampton, UK; 30 Division of Research, Greenwood Genetic Center, Greenwood, SC, USA; 31 Molecular, Cellular, and Genomic Biomedicine Group, Valencia, Spain; 32 University and Polytechnic La Fe Hospital of Valencia, Valencia, Spain; 33 The Ruth & Bruce Rappaport Faculty of Medicine, Technion-Israel Institute of Technology, Haifa, Israel; 34 Manchester Centre for Genomic Medicine, Saint Mary’s Hospital, Manchester University NHS Foundation Trust, Manchester, UK; 35 Division of Evolution, Infection and Genomics, School of Biological Sciences, Faculty of Biology, Medicine and Health, University of Manchester, Manchester, UK; 36 Biochemistry and Biophysics Center, National Heart, Lung and Blood Institute, Bethesda, MD, USA; 37 Centre for Gene Therapy and Regenerative Medicine, King’s College London, London, UK

**Keywords:** Inherited retinal diseases, IRD, macular dystrophy, KATNA1, katanin, severases, microtubules, microtubule-severing

## Abstract

Inherited retinal diseases (IRDs) encompass a broad spectrum of genetic conditions leading to visual impairment. In this study, we identify *KATNA1*, encoding the catalytic p60 subunit of the microtubule-severing enzyme katanin, as a previously unrecognized cause of autosomal dominant macular dystrophy (adMD), a form of IRD. Specifically, we could ascertain the presence of 10 heterozygous missense changes affecting six conserved amino acids in 21 individuals from 16 unrelated families from various parts of the world, all presenting with non-syndromic MD of variable severity. Structure-guided analyses indicated that the identified variants potentially disrupt katanin’s assembly into hexamers or its ability to bind or hydrolyze ATP, thus compromising its microtubule-severing function. Characterization of patient-derived fibroblasts revealed accumulation of acetylated microtubules both in the cytoplasm and within the primary cilium, together with an altered subcellular distribution of KATNA1. Immunostaining of human retinal tissue showed that KATNA1 specifically localizes to photoreceptors, with distinct distribution patterns between rod and cone photoreceptors. Immunogold transmission electron microscopy confirmed this finding, revealing KATNA1 distribution along the rod axoneme and predominantly within the cone connecting cilium. Together, these results establish *KATNA1* as a novel gene associated with adMD, possibly accounting for ~4% of all unresolved MD cases, and associate defective microtubule severing and cytoskeletal dysregulation with macular degeneration.

## Introduction

Inherited retinal diseases (IRDs) are a genetically and clinically heterogeneous group of Mendelian disorders that typically cause visual impairment through the degeneration or dysfunction of photoreceptors (rods and cones), and/or the retinal pigment epithelium (RPE) cells,^1,2^ collectively playing essential roles in phototransduction, visual signal processing, and homeostatic support of the neural retina.^3,4^ IRDs are typically classified according to the cell type that is predominantly affected - such as rod-cone dystrophy (retinitis pigmentosa) or cone-rod dystrophy. Other IRD forms are defined by the region of the retina that is impacted by the disease. For instance, macular dystrophies (MDs) are characterized by the loss or dysfunction of cells in the macula, the central retinal region enriched in cone photoreceptors and essential for high-resolution, daylight, and color vision. Consequently, individuals with MD commonly experience central vision loss, with associated symptoms involving disturbance of color sensitivity or visual distortion. However, the age of onset, the range of experienced symptoms, and the rate of their progression can vary markedly among affected individuals, often depending on the underlying genetic cause.^5,6^

To date, more than 500 genes have been associated with IRDs.^7^ However, missing heritability still remains relatively high, with a significant proportion of affected individuals (up to 50%, depending on the ethnicity, represented phenotypes, suspected mode of inheritance, and testing methods used) lacking a molecular diagnosis.^8–12^ In particular, MDs have been linked to only 30 genes, of which half display an autosomal dominant inheritance pattern (adMD) and are involved in diverse biological processes, including transcriptional regulation, visual cycle and phototransduction, lipid metabolism, or cell adhesion,^7^ underscoring the complex molecular mechanisms required to preserve macular integrity and function.^13^

Among the cellular systems critical to retinal architecture and photoreceptor homeostasis, the microtubule cytoskeleton and its associated regulators play particularly important roles. In photoreceptors, microtubules form a polarized scaffold extending from the inner segment (IS) through the connecting cilium (CC) into the outer segment (OS) axoneme.^14^ They not only provide structural stability to the CC and OS^15,16^ but also facilitate the bidirectional protein trafficking between the IS and OS via intraflagellar transport,^17^ support synaptic function,^18^ and drive ciliogenesis.^19,20^ The dynamic remodeling of microtubules, through regulated polymerization, depolymerization, and severing, is essential in all eukaryotic cells for diverse cellular processes, including cilia biogenesis, cell division, phototropism, and neurogenesis.^21,22^ In the retina, dysregulation of these processes via altered polymerization dynamics,^23^ defective motor proteins,^24^ or imbalanced tubulin post-translational modifications^25–27^ has been implicated in several pathologies, ranging from isolated IRDs to syndromic diseases, affecting other organs and systems.^28^ However, a direct link between microtubule-severing impairment and IRDs has not previously been established.

Microtubule-severing enzymes (or severases), including spastin and katanin, orchestrate cytoskeletal remodeling by breaking microtubules into shorter polymers, regulating general microtubule mass, enabling the dynamic reorganization of cellular architecture, and facilitating intracellular transport.^22^ Katanin consists of a catalytic p60 subunit (KATNA1, MIM: 606696), which belongs to the AAA+ (ATPases Associated with diverse cellular Activities) family, and a regulatory p80 subunit (KATNB1, MIM: 602703).^29^ The enzyme assembles into a hexamer with a central ring-shaped structure formed by the KATNA1 subunits.^22,30^ KATNB1 binds peripherally to modulate catalytic activity, stability, and subcellular localization of the complex.^31^ Upon ATP binding and hydrolysis, conformational changes within the hexamer generate a mechanical force that extracts tubulin dimers from the microtubule lattice, leading to filament destabilization and severing.^30,32^ While biallelic variants in *KATNB1* have been linked to a neurodevelopmental disorder characterized by microcephaly and cortical malformations (MIM: 616212),^33^ the involvement of *KATNA1* in human Mendelian disease has not previously been described.

In this study, we identify ten distinct heterozygous missense variants in *KATNA1* as a previously unrecognized cause of a non-syndromic IRD in 21 affected individuals from 16 unrelated families. All affected subjects presented with a distinct form of MD with predominant cone involvement. Structural modelling showed that the identified *KATNA1* variants cluster within highly conserved residues of katanin and are predicted to impair its ATP-dependent microtubule-severing activity. Analysis of a patient-derived cell line revealed accumulation of acetylated microtubules in both the cytoplasm and primary cilia, together with aberrant subcellular distribution of KATNA1. Furthermore, immunofluorescence and electron microscope analysis of human retina demonstrated specific localization of KATNA1 to photoreceptors, with distinct distribution patterns between rods and cones. Together, these findings not only establish *KATNA1* as a novel causative gene for adMD but also highlight a previously understudied role of microtubule severing in the maintenance of macular structure and function, opening new avenues for further studies and potential therapeutic intervention.

## Results

### Clinical features of the affected individuals

**Figure 1.**
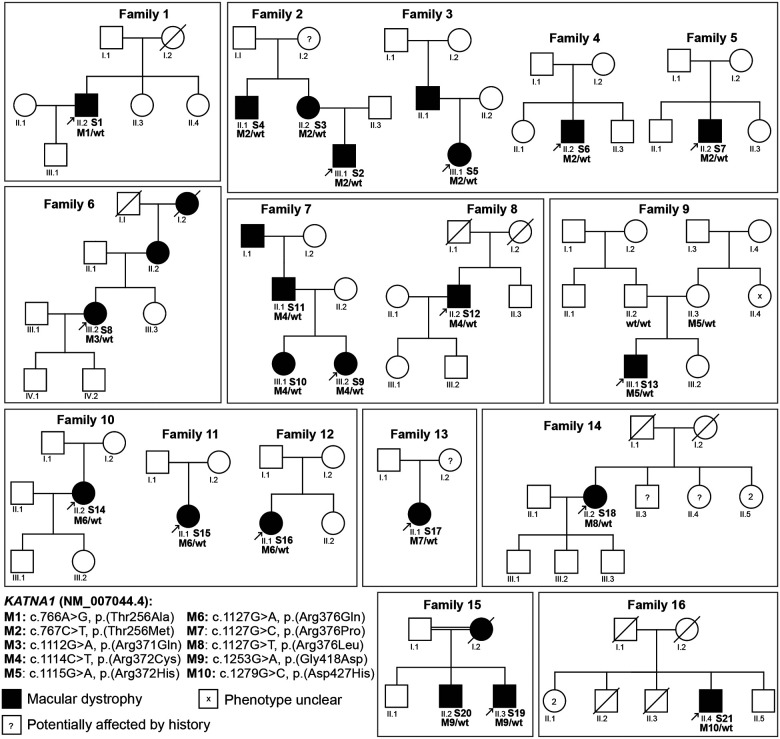
Pedigrees of the families carrying *KATNA1* variants identified in this study. Disease-associated alleles are indicated by MX (corresponding to listed variants M1–M10), while “wt” denotes the wild-type allele. Families are grouped according to the identified *KATNA1* variant, and probands are marked by arrows.

A total of 21 affected individuals from 16 unrelated families were ascertained through ophthalmic examination (Figure 1, Table 1). Most were reported as isolated cases, whereas autosomal dominant inheritance was documented in five families (Families 2, 3, 6, 7, and 15). All participants exhibited ocular findings consistent with non-syndromic macular dystrophy, with detailed clinical data summarized in Table S1.

Typical symptoms included reduced visual acuity, metamorphopsia, and light sensitivity, while five subjects additionally reported night vision difficulties (S5, S7, S14, S18, and S19). The age of onset was highly variable, ranging from 10 to 61 years, while in three cases (S5, S7, and S11) this could not be reliably established (Table 1). Notably, eight individuals reported no subjective symptoms at their most recent examination (ages 7 to 67 years). Although most of those cases were younger, two individuals (S3 and S4, Family 2) did not experience disease symptoms at later ages (62 and 67 years, respectively), highlighting the variable expressivity of this disorder (Table 1). Decimal best corrected Snellen visual acuity (BCVA) ranged from 1.25–1.0 in subjectively asymptomatic or early-stage cases to 0.1–0.5 in more advanced disease. The progressive nature of macular involvement was illustrated by the BCVA decline in two subjects (S1 and S8) for whom longitudinal data were available (Table S1).

**Figure 2.**
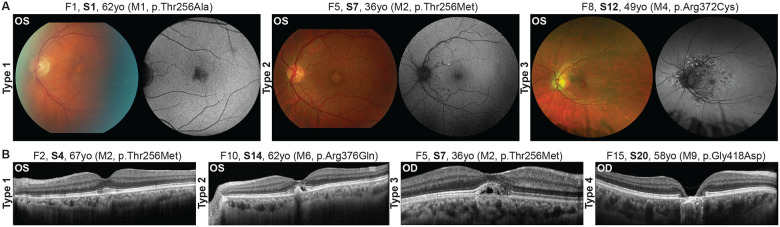
Multimodal retinal imaging of representative types of *KATNA1*-associated macular dystrophy. **A.** Fundus images of the left eye from three individuals, illustrating Type 1 (left), Type 2 (middle), and Type 3 (right) forms of macular dystrophy described in this study. Color or pseudocolor fundus images are shown on the left of each pair, with corresponding fundus autofluorescence images (FAF) on the right. **B.** Macular spectral-domain optical coherence tomography (OCT) images from four individuals, representing OCT Types 1 through 4 of the disease. Description above each panel indicates the family (FX), subject (SX), age at examination (yo - years old), and identified variant (MX) with its protein notation. OS - left eye; OD - right eye.

Based on fundus imaging, MD could be broadly categorized into three phenotypic types (Table S1, Figure 2A): Type 1, lesions confined to the macula, characterized by foveolar drusen-like deposits and/or macular and perimacular deposits; Type 2, features of Type 1 plus posterior pole involvement within the vascular arcades; Type 3, features of Type 2 plus macular degeneration extending to the nasal retina of the optic disc. The disease was bilateral in all cases and largely symmetrical, with the exception of three individuals (S1, S9, and S14) who showed mild interocular asymmetry (Table S1).

Macular optical coherence tomography (OCT) revealed irregularities and thickening of the ellipsoid zone and RPE. The observed OCT abnormalities could be grouped into four clinical types (Table S1, Figure 2B): Type 1, thickening of the ellipsoid zone and/or pseudovitelliform deposits; Type 2, pseudovitelliform eruptive lesions; Type 3, localized disruption or loss of outer retinal layers at the fovea (OCT gap); and Type 4, generalized atrophy. In six individuals, OCT changes were asymmetrical, with greater involvement in one eye, whereas the remaining subjects exhibited symmetrical patterns between both eyes (Table S1).

Full-field electroretinography (ERG) was available for six subjects (Table S1), all of whom demonstrated a detectable signal. In four individuals (S7, S13, S14, and S18), ERGs were within normal limits despite different disease advancement. Only two siblings from Family 15 (S19 and S20, corresponding to bilateral fundus Type 1 and Type 3, respectively) exhibited mild scotopic and photopic abnormalities. Overall, these findings indicate that peripheral retinal function was largely preserved in this cohort.

Notably, disease classification based on fundus images did not consistently correspond to OCT types, particularly in more advanced disease cases where several individuals with fundus Type 3 remained OCT Type 1 (S6, S11, S12, S16, S21). OCT types generally correlated with BCVA: individuals with Type 1 OCT typically maintained good visual acuity (0.8–1.25), apart from two subjects (S17 and S21) who experienced visual decline despite relatively preserved OCT structure (Table S1). As expected, more advanced OCT types were associated with lower BCVA. No consistent pattern of refractive error was identified across the cohort. Similarly, no additional ocular or systemic features were repeatedly observed. A few subjects presented with common conditions, including arterial hypertension, asthma, osteoporosis, pancreatic cysts, and infantile eczema, which were likely unrelated to the Mendelian MD (Table 1).

Finally, given the late onset and frequent asymptomatic disease presentation, it is possible that some relatives reported as unaffected in Figure 1 (who were neither clinically examined nor genetically tested) may still be at risk of developing the condition later in life.

### Molecular analysis and identification of KATNA1 variants

All probands included in this study were previously tested negative for causative variants in known IRD-associated genes. Analysis of sequencing data from individuals with a clinical diagnosis of MD, using a customized analytical pipeline focused on rare variants in shared genes, led to the identification of heterozygous variants in *KATNA1* in 21 affected members from 16 unrelated families (Figure 1, Table 1).

**Figure 3.**
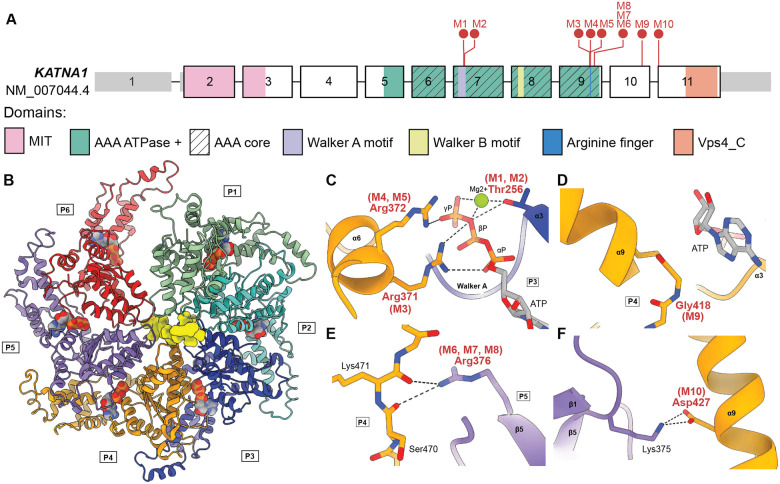
Mutational landscape and structural modeling of *KATNA1* variants. **A.** Schematic representation of *KATNA1* (based on its canonical transcript, NM_007044.4) showing the positions of variants identified in this study (M1–M10). Exons are represented by numbered boxes, whereas introns as a horizontal line (not to scale). Untranslated regions (UTRs) are shown in grey. Functional domains are color-coded according to the legend. Abbreviations: MIT - Microtubule interacting and trafficking; AAA ATPase + - ATPase associated with diverse cellular activity; Vps4_C - Vacuolar protein sorting-associated protein 4. All variants clustered in exons 7, 9, 10, and 11, with the majority localized within the AAA domain (M1–M8). **B.** Atomic model of the *C. elegans* katanin hexamer (PDB ID: 6UGD).^32^ Protomers (P1–P6) are color-coded: P1, green; P2, cyan; P3, blue; P4, orange; P5, purple; P6, red. A polyglutamate substrate peptide within the katanin pore is displayed as a yellow surface. **C-F.** Close-up views of amino acid residues (in red, bold) affected by respective KATNA1 variants (MX) identified in this study. Hydrogen bonds are shown as black dashed lines. P - respective phosphates of ATP (Pα, Pβ, and Pγ).

In total, we identified ten distinct single-nucleotide substitutions, all resulting in missense alterations (Table S2). These variants clustered within exons 7, 9, 10, and 11 of *KATNA1*, with the majority of them affecting residues within the AAA ATPase domain (AAA domain), which is crucial for microtubule severing activity and ATP-dependent oligomerization (Figure 3A).^32^ All altered amino acids were highly conserved across orthologs in vertebrates, invertebrates, plants, and unicellular eukaryotes (Figure S2A) with a GERP++ score greater than 4.9 (range: −12.3 to 6.17)^34^. They were predicted to be deleterious by multiple *in silico* tools, including MutScore (scores between 0.605 and 0.967),^35^ AlphaMissense (scores between 0.897 and 0.999),^36^ and REVEL (scores between 0.846 and 0.990)^37^ (maximum score of 1.000 for each tool). All identified variants were either absent from or extremely rare across multiple population databases, such as gnomAD,^38^ The All of Us Research Program,^39^ and GenomeAsia 100k,^40^ with no reported homozygotes. According to the current guidelines for variant classification, the majority of these novel changes were assessed as pathogenic or likely pathogenic (Table S2).^41^

In Family 1 from Germany, proband S1, the only affected individual, was found to carry a heterozygous *KATNA1* variant c.766A>G, p.(Thr256Ala) (M1; reference sequences for *KATNA1* mRNA and protein: NM_007044.4 and NP_008975.1, respectively), located within the Walker A motif (Figure 3A) of the AAA domain, known for its interaction with the phosphate groups of an ATP. This variant was not observed in the healthy population, based on the two versions of the gnomAD database (v2.1.1 and v4.1.0), as well as the All of Us repository (Table S2).

Family 2, of South-East Asian ancestry (Bangladesh), comprised three individuals with clinically confirmed MD across two generations (S2, S3, and S4), consistent with autosomal dominant inheritance (Figure 1). They all carried the heterozygous *KATNA1* variant c.767C>T, p.(Thr256Met) (M2), which altered the same amino acid residue affected by M1. While absent from gnomAD v2, this variant was present in three individuals of European descent in gnomAD v4 (aged 45–70 years), suggesting the possibility of subclinical disease manifestations and underdiagnosis (Table S2). Interestingly, the same M2 variant was also identified in several unrelated MD cases. These included an individual S5 from a family of Russian origin with a history of MD (Family 3), as well as two isolated cases from Europe: S6 (Family 4, from Germany), and S7 (Family 5, ascertained in the United Kingdom). Notably, subject S6 was asymptomatic at the last follow-up (at 31 years old), whereas individuals S5 and S7 were diagnosed at 35 and 36 years of age, respectively, although the precise age at symptom onset was undocumented (Table 1). In Families 4 and 5, segregation analysis and clinical evaluation of reportedly unaffected parents were not possible (Figure 1); thus, the possibility of asymptomatic carrier status could not be excluded.

Family 6 from Germany included three reported generations of affected individuals with MD, yet only the proband (S8) was available for genotyping (Figure 1). She presented with first symptoms in her fourth decade of life and harbored the heterozygous c.1112G>A, p.(Arg371Gln) variant (M3), absent from the gnomAD database. This change affected a very conserved arginine at position 371, located in the AAA domain (Figure 3A) and serving a specific role in ATP hydrolysis (see below).

Family 7 was a pedigree of South Asian origin (Indian Gujarati) and included three reportedly affected generations (Figure 1). The proband (S9), a 7-year-old female who was asymptomatic at her last examination, had been referred for ophthalmic evaluation following a routine optician visit that revealed suboptimal vision and macular changes. She was found to carry the heterozygous *KATNA1* variant c.1114C>T, p.(Arg372Cys) (M4). This residue, located immediately adjacent to Arg371, is also within the AAA domain and is known as the KATNA1 arginine finger (Figure 3A). The variant was not observed in the control population and co-segregated with disease in her affected older sister (S10) and father (S11) (Figure 1). Interestingly, this variant was independently identified in an unrelated European individual from Germany (S12, Family 8), who developed MD symptoms at the age of 48 (Table 1). Furthermore, the same codon was found to be altered in proband S13 from Family 9. This 6-year-old boy of British descent carried the c.1115G>A, p.(Arg372His) variant (M5), which was predicted to be highly deleterious (Table S2). It was absent from gnomAD v2; however, it was identified in two European individuals in gnomAD v4. Segregation analysis revealed that this variant was inherited from the mother (F9:II.3), who remained unaffected at the age of 44 years (Figure 1), as confirmed by her ophthalmic examination, including FAF and OCT. Notably, the maternal aunt (F9:II.4) exhibited a macular lesion in the right eye during an assessment performed in 2004 at the age of 29; however, she was unavailable for further genotyping or clinical assessment (Figure 1).

Individuals from Families 10, 11, and 12, originating from Germany, Israel (Ashkenazi-Yemenite ancestry), and the United Kingdom (of Iranian descent), respectively, were all ascertained as isolated cases (S14, S15, and S16) with reportedly unaffected parents unavailable for further genetic testing (Figure 1). All three individuals carried the same recurrent heterozygous *KATNA1* variant c.1127G>A, p.(Arg376Gln) (M6), located within the AAA domain (Figure 3A). This substitution was observed in one European individual in the gnomAD dataset (Table S2). Subjects S14 and S16 developed disease symptoms at a relatively early age (18 and 27 years, respectively), whereas individual S15 was asymptomatic at her most recent examination at the age of 9 years old (Table 1). Interestingly, in Family 13, of British ancestry, the proband (S17) carried a different heterozygous missense change affecting the same nucleotide as M6: c.1127G>C, p.(Arg376Pro) (M7), which was also reported in one European individual in gnomAD v4 (Table S2). At her last examination, at age 29 years, S17 was subjectively asymptomatic. Notably, her mother was reported to have visual problems; however, she was unavailable for clinical assessment or genetic testing (Figure 1). Furthermore, another heterozygous variant at the same nucleotide position, c.1127G>T, p.(Arg376Leu) (M8), was identified in proband S18 from Family 14, of Turkish descent. This individual developed MD at the age of 20 years, and had two younger siblings with reported macular vision problems, who were not currently genetically tested or clinically assessed (Figure 1). This variant was not found in the queried population databases (Table S2).

Family 15 was a consanguineous Ashkenazi Jewish pedigree from Israel, in which the parents were first-degree cousins and two successive generations were reportedly affected with MD (Figure 1). The two genotyped brothers (S19 and S20) both carried a heterozygous variant outside of known annotated functional domains of *KATNA1* c.1253G>A, p.(Gly418Asp) (M9), which was not identified in the control population (Table S2). In addition to MD, they were reported to have mild bilateral hearing loss and variable concomitant extra-ocular symptoms, including bronchial asthma, osteoporosis, hydronephrosis, and mitral valve prolapse (Table 1). These additional findings may have an unrelated genetic cause, possibly due to the reported consanguinity in the family.

Subject S21 was an isolated case of late-onset MD from the Spanish Family 16 (Figure 1). He was found to carry a heterozygous missense in the last coding exon of *KATNA1* (exon 11): c.1279G>C, p.(Asp427His) (M10). This change was likewise not found in any population reference datasets (Table S2).

Remarkably, all ten identified variants affected only six distinct amino acid residues. This recurrent targeting of Thr256, Arg372, and Arg376 by independent nucleotide substitutions across multiple unrelated individuals supported their functional relevance and mutational sensitivity, as further illustrated by the pathogenicity landscape in the *in silico* KATNA1 protein model (Figure S2B).

### Structure-guided analysis of KATNA1 variants

Katanin is a hexameric AAA ATPase that assembles into a ring-shaped complex to mediate microtubule severing through ATP hydrolysis. Six protomers (P1–P6) of its catalytic p60 subunit, KATNA1, oligomerize to form the functional pore of the hexamer (Figure 3B), which then engages the microtubule lattice and extracts tubulin dimers in an ATP-dependent manner.^32,42^ The function of the hexamer is regulated by accessory proteins, including the regulatory p80 subunit (KATNB1) and KATNIP (MIM: 616650), which facilitate anchoring, regulation, and subcellular localization of the complex.^43,44^

As in other AAA ATPases, the nucleotide-binding pocket of katanin is located at the protomer-protomer interface, where ATP phosphates are coordinated *in trans* by two conserved arginine residues of the neighboring protomer. As we previously demonstrated, in KATNA1, these correspond to arginines at positions 371 and 372.^30,32^ Arg371 coordinates the α- and β-phosphates of ATP, while Arg372 (known as the ‘arginine finger’, Figure 3A) interacts with the γ-phosphate, both essential for ATP hydrolysis.^32^ We therefore performed structure-guided modeling of all variants identified in this study. The Arg371Gln substitution (M3) is predicted to impair ATP coordination and disrupt hydrolytic activity, thereby compromising microtubule severing (Figure 3C). Similarly, the two changes of the adjacent arginine, Arg372Cys (M4) and Arg372His (M5), are expected to impair ATP hydrolysis while preserving oligomerization and microtubule binding (Figure 3C). Thus, we predict that these three variants (M3, M4, and M5) are expressed but result in a catalytically impaired enzyme that is either completely inactive or severely defective in its severing function.

Based on our previous structural and functional data, we also predict that all the other identified variants (M1–M2, M6–M10) are likely to interfere with KATNA1 oligomerization, with downstream effects on microtubule binding and severing.^30,32^ Specifically, the Thr256 residue, located within the Walker A motif (Figure 3A), plays a critical role in coordinating ATP via hydrogen bonding. The Thr256Ala variant (M1) abolishes these interactions, disrupting ATP and Mg^2+^ binding (Figure 3C). The Thr256Met substitution (M2), which replaces a small polar residue (threonine) with a bulkier non-polar methionine, is predicted to create steric hindrance that interferes with nucleotide binding (Figure 3C). Similarly, the Gly418Asp variant (M9) introduces a negatively charged side chain into a conserved loop connecting α-helices 8 and 9 and is predicted to destabilize ATP binding and therefore hexamer assembly (Figure 3D). The Arg376 creates hydrogen bonds with residues Ser470 and Lys471 in the adjacent protomer. Its substitution to glycine (Arg376Gly, M6) would disrupt these bonds, interfering with hexamer assembly. Its substitution to proline (Arg376Pro, M7) would likewise destabilize this oligomerization interface, as would substitution into leucine (Arg376Leu, M8) (Figure 3E). Lastly, Asp427, positioned at the protomer interface, forms a salt bridge with Lys375. A substitution of aspartic acid to histidine at this position (Asp427His, M10) would abolish this interaction, compromising inter-protomer contacts and hexamer stability (Figure 3F).

To assess the homology of the highly conserved AAA domain^45^ of KATNA1 and another microtubule-severing enzyme, spastin (SPAST, MIM: 604277), we aligned their sequences (Figure S2C). All pathogenic missense substitutions in *SPAST* that cause autosomal dominant spastic paraplegia type 4 (MIM: 182601) cluster within this domain, underscoring its functional importance.^46^ Our analysis showed that the majority of KATNA1 variants identified here map to homologous positions of pathogenic SPAST alleles (Figure S2C). Specifically, M4 (p.Arg372Cys) corresponds to SPAST p.Arg499Cys (ClinVar ID: 5660), which affects the arginine finger, impairing ATP hydrolysis, but not ATP binding, and thus leading to constitutive microtubule binding and bundle formation.^47,48^ Similarly, M1, M5, and M6 exactly mirror established pathogenic *SPAST* variants (ClinVar IDs: 188190, 240950, 1992311), while M2, M7, and M8 represent different substitutions of their corresponding residues (ClinVar IDs: 2767964, 219575).

In summary, our structural modeling indicates that the majority of variants identified in this study destabilize hexamerization of KATNA1 or impair ATP binding, whereas three variants (M3, M4, and M5) are predicted to directly disrupt ATP hydrolysis through substitution of conserved arginine residues, consistent with homologous mutations reported in the other microtubule severing enzyme, spastin. Regardless of the precise molecular mechanism, all detected variants are expected to compromise the microtubule severing activity of katanin.

### Effect of recurrent KATNA1 variant in the patient-derived cell line

To experimentally assess the cellular consequences of predicted impairment of katanin function, we analyzed a patient-derived fibroblast cell line obtained from individual S14, who is heterozygous for the recurrent *KATNA1* variant M6, targeting Arg376 and expected to disrupt hexamer assembly.

**Figure 4.**
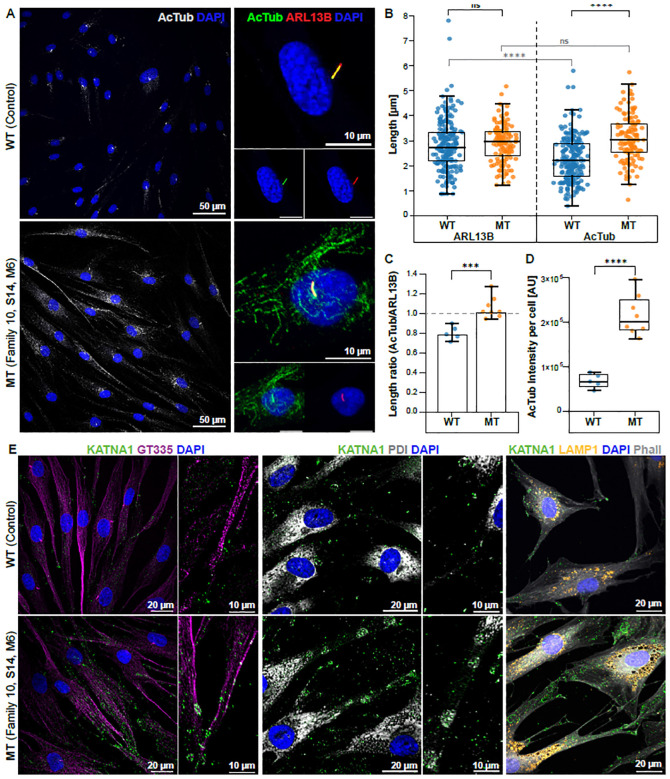
Cellular consequences of *KATNA1* variant in a patient-derived fibroblast cell line. Serum-deprived fibroblasts derived from individual S14 (MT), heterozygous for the KATNA1 M6 variant (p.Arg376Gln), were compared with healthy control fibroblasts (WT). **A.** Left panel: widefield images showing acetylated tubulin (AcTub); right panel: representative high-magnification images of individual ciliated cells labeled for AcTub (green) and the ciliary member marker (ARL13B, red). Merged images show overlap between the two markers (yellow). **B.** Quantification of primary cilium lengths in WT and MT fibroblasts using both AcTub and ARL13B as markers, from three technical replicates. ARL13B: n = 224 (WT), n = 132 (MT); AcTub: n = 221 (WT), n = 120 (MT); where n denotes the number of cilia, pooled across replicates. **C.** Ratio of AcTub-positive to ARL13B-positive ciliary lengths, calculated from matched images [n = 5 (WT), n= 8 (MT), where n denotes the number of images]. **D.** Quantification of total AcTub fluorescence intensity per image [n = 5 (WT), n= 8 (MT)], normalized to the number of cells. AU - arbitrary fluorescence units. Median values are indicated by horizontal thick bars; for box plots, standard notation was applied. Statistical significance is indicated as follows: ns, not significant (p-value > 0.05); ***, p-value ≤ 0.001; ****, p-value < 0.0001. **E.** Representative immunofluorescence images showing KATNA1 localization (green) in WT and MT fibroblasts, in combination with markers of polyglutamylated tubulin (GT335, magenta; left), endoplasmic reticulum (PDI, grey; middle), and lysosomes (LAMP1, orange; right). Nuclei were stained with DAPI (blue), and the actin cytoskeleton was visualized with phalloidin (Phall) in the right panel. Scale bars are indicated in the respective images.

As katanin has been shown to play a role in ciliogenesis and cilia resorption,^49^ we first assessed the formation and morphology of the primary cilium using ARL13B as a marker of the ciliary membrane (Figure 4A). This analysis revealed no significant differences in overall ciliation rate following serum deprivation (not shown) or in the length of protruding ciliary membrane between mutant (MT) and control (WT) fibroblasts (p-value = 0.357, Figure 4B). However, staining for acetylated tubulin (AcTub), which directly identifies a population of stable microtubules, showed that MT cells displayed significantly increased ciliary lengths (p-value < 0.0001, Figure 4B). These data were further validated by the calculated ratio between AcTub- and ARL13B-related lengths, showing statistically significant higher values in MT cells (average: 1.06) vs. WT (average: 0.8). This suggested that in MT cells AcTub extended along the entire ciliary length, consistent with an increased axonemal microtubule stability related to impairment of katanin function (p-value = 0.0003, Figure 4C). We then expanded this analysis to the cytoplasmic microtubule network and observed a marked increase in overall AcTub immunofluorescence signal in MT fibroblasts compared to WT (Figure 4A). This was further confirmed by quantitative measurement of total AcTub fluorescence intensity per cell (background-corrected average in MT: 2.15×10^5^, in WT: 0.68×10^5^; p-value < 0.0001, Figure 4D) and was consistent with previously described cellular defects resulting from impaired spastin-mediated microtubule severing.^50,51^

In parallel, KATNA1 displayed a strikingly altered subcellular distribution in MT fibroblasts. Whereas control cells exhibited a sparse punctate KATNA1 pattern, MT cells showed prominent swirl-like KATNA1-positive structures, predominantly localized peripherally relative to the nucleus (Figure 4E). These aggregates aligned along GT335-positive microtubules, consistent with the established association of katanin-mediated severing with glutamylation of tubulin.^52^ Notably, KATNA1-positive structures were also directly associated with the distal endoplasmic reticulum (ER) network, as marked by colocalization with protein disulfide isomerase (PDI), suggesting aberrant recruitment or retention of KATNA1 at specific microtubule subpopulations and ER-associated sites. In addition, MT fibroblasts exhibited enlarged lysosomal compartments (labeled by LAMP1) compared to WT (Figure 4E), likely reflecting secondary disruption of cellular homeostasis arising downstream of impaired microtubule dynamics.^53^

Overall, these findings demonstrate that, at the cellular level, the M6 variant of KATNA1 leads to increased acetylation of both ciliary microtubules (~100% of their length vs 80% in the control) and the general cellular tubulin population, together with an aberrant subcellular distribution of KATNA1, consistent with its predicted disruption of katanin-mediated microtubule severing.

### Expression and localization of KATNA1 in the human retina

As a microtubule-severing enzyme, KATNA1 is expected to localize to microtubule-rich compartments in the human body. Indeed, expression datasets indicate KATNA1 enrichment in ciliated cells,^54^ with the highest levels observed in testis, followed by thymus, skeletal muscle, and retina (Figure S3).^55–57^ Single-cell RNA-seq data (scRNA-seq) from human retina revealed a relatively low and uniform expression of *KATNA1* across all cell types, including cells from the RPE.^58,59^

**Figure 5.**
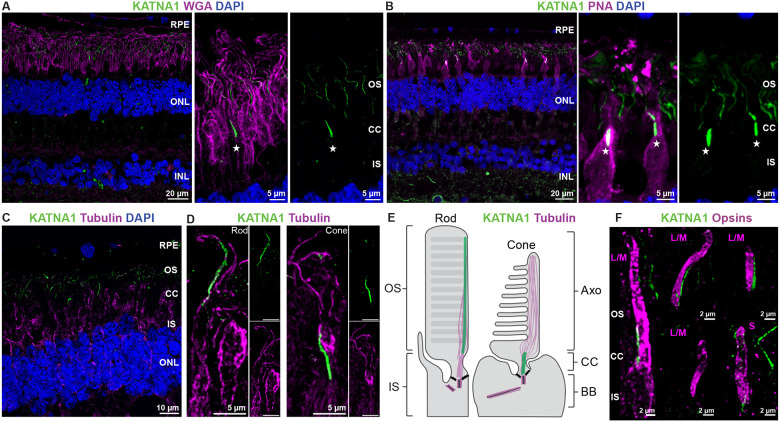
Immunofluorescence imaging of KATNA1 in the human retina. **A-B.** Human retina sections stained with KATNA1 (green) together with WGA (labeling rod photoreceptors, magenta, A) or PNA (labeling cone photoreceptors, magenta, B), respectively. KATNA1 displays distinct localization patterns in rods versus cones: in rods, it is enriched in the outer segments, whereas in cones it concentrates at the connecting cilium. In the higher-magnification images cones are marked with white stars. **C-D.** Co-staining of KATNA1 (green) and tubulins (magenta). Panel D shows higher magnification views depicting a single rod and cone, illustrating the differences in KATNA1 localization. **E.** Schematic representation of rod and cone photoreceptors’ morphology summarizing the observed KATNA1 distribution (green) with respect to tubulin (magenta). **F.** KATNA1 (green) staining with markers of distinct cone opsins (magenta) for L/M and S cones, showing its localization across cone subtypes. Nuclei were counterstained with DAPI (blue). Scale bars are indicated in the respective images. Abbreviations: RPE - retinal pigment epithelium; OS - outer segments; IS - inner segments; Axo - axoneme; CC - connecting cilium; BB - basal body; ONL - outer nuclear layer; INL - inner nuclear layer.

To further investigate the presence and localization of KATNA1 in the human retina, we performed immunofluorescence staining on paraffin-embedded (FFPE) retinal sections. This revealed specific KATNA1 immunoreactivity in the photoreceptor layer (Figure 5), with distinct localization patterns in rods and cones. In rods, stained by Wheat Germ Agglutinin (WGA), KATNA1 was detected throughout the length of the OS (Figure 5A and E). Consistent with its association with the microtubule cytoskeleton, it partially colocalized with the tubulin signal (α- and β-tubulins, Figure 5C) in the rod axoneme, with its signal continuing distally into the OS (Figure 5D and E). In contrast, in cones - labeled by Peanut Agglutinin (PNA) - KATNA1 signal was restricted mainly to the CC and did not extend further into the OS (Figure 5B, D, and E). Notably, KATNA1 was not uniformly detected across all cones (Figure 5B). Using different opsins as markers to test for cone subtype specificity, we observed a clear KATNA1 signal in only a subset of L/M cones, whereas it was consistently present in all S cones, albeit with a more dispersed pattern (Figure 5F).

**Figure 6.**
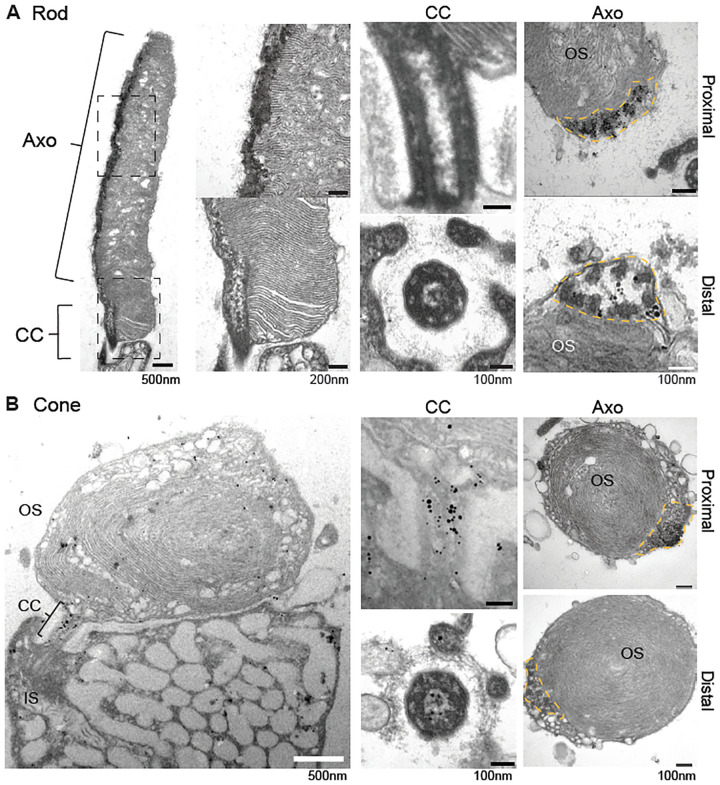
Immunogold transmission electron microscopy (TEM) of KATNA1 in the human retina. **A.** TEM images of rod photoreceptors. KATNA1 is present along the axoneme (Axo) within the outer segment (OS) but is absent from the connecting cilium (CC). Higher-magnification longitudinal and transverse views demonstrate a high density of KATNA1 immunogold labeling both at the proximal and distal regions of the rod OS, in close association with axonemal microtubules. **B.** Same as in A, but for cone photoreceptors. KATNA1 presence is restricted to the CC and the proximal OS. Within the CC, it localizes specifically to the lumen, surrounded by microtubule doublets. Dense labeling is also observed in the proximal axoneme, whereas KATNA1 is absent from the cone OS. Axonemal structures are delineated by yellow dashed lines in the transverse close-up views. Scale bars are shown below the respective panels. Abbreviations: Axo - axoneme; CC - connecting cilium; OS - outer segments; IS - inner segment.

We next assessed the subcellular distribution of KATNA1 at ultrastructural resolution using immunogold transmission electron microscopy (TEM) of the photoreceptors from healthy human retina. In rods, KATNA1 labeling was absent from the CC and instead localized specifically along the axoneme (Figure 6A), consistent with the immunofluorescence data. Labeling extended along the entire length of the OS axoneme, with a high density even at its distal end, where cross-sections showed KATNA1 closely associated with microtubules (Figure 6A). In cones, the signal was conversely detected mostly in the CC and proximal OS (Figure 6B), although the cone subtype could not be determined in this analysis. Transverse views revealed KATNA1 labeling predominantly within the cone CC lumen, surrounded by microtubule doublets (Figure 6B).

Since scRNA-seq datasets also showed a potential expression of *KATNA1* in the RPE, we assessed FFPE sections containing intact human RPE, as well as monolayers of induced pluripotent stem cell-derived RPE (iPSC-RPE) for the presence of KATNA1 protein. Immunostaining revealed no specific KATNA1 signal in this tissue (Figure S4A) or in the iPSC-RPE cells, at various maturation stages (Figure S4B). We could only detect sparse punctate KATNA1 labeling at week one of iPSC-RPE maturation, which partially colocalized with polyglutamylated tubulin (recognized by GT335). This staining suggests KATNA1 presence at the tip of retained primary cilia (Figure S4B), which are known to support early iPSC maturation and polarization.^60,61^

Taken together, these findings demonstrate a highly specific localization of KATNA1 in photoreceptors of the human retina, with distinct patterns in rods and cones. Its enrichment at the cone CC and proximal OS, in contrast to its localization along the rod axoneme, points to a compartment-specific role of KATNA1 within different types of photoreceptors.

## Discussion

Understanding the molecular basis of IRDs is essential not only for accurate diagnosis and prognosis, but also for effective genetic counselling of families and the development of novel therapeutic strategies.^62^ However, despite major advances in Next-Generation Sequencing and associated analytical pipelines, establishing a molecular diagnosis for many rare diseases, including IRDs, remains challenging.^63–66^ Within the IRD spectrum, macular dystrophies (MDs) represent a clinically significant subgroup, yet they remain comparatively underrepresented at the genetic level, with a substantial fraction of MD cases still lacking a defined molecular cause.^67^ This persistent diagnostic gap is attributed to several factors, including technical limitations in detecting non-coding and structural variants,^68^ challenges in variant interpretation,^69^ and the existence of disease-causing genes and mechanisms yet to be identified. In recent years, the characterization of new genes underlying both dominant and recessive MD has expanded the repertoire of cellular processes recognized as critical for macular integrity, while also defining novel MD clinical subtypes.^70–74^ Nevertheless, our understanding of the molecular mechanisms that sustain macular function remains incomplete, in part reflecting the shortcomings of commonly used animal and cellular models, which fail to recapitulate macula-specific features.^75,76^

The macula, a retinal region with unique morphology, contains the highest density of cones, enabling high-acuity daytime vision.^77^ Accordingly, patients with MD typically present with common initial symptoms, such as central vision disturbance or scotomas.^5^ However, MD encompasses a highly heterogeneous clinical spectrum,^5^ ranging from severe and rapidly progressive diseases,^78^ to late-onset,^79^ or even asymptomatic presentations detected incidentally.^80^ Therefore, the global prevalence of MD (approximately 1 in 10,000),^5^ may be underestimated due to mild or individually variable symptoms and clinical overlap with common conditions such as age-related macular degeneration (AMD).^81–83^ These factors underscore the importance of genotyping individuals with MD-like presentations, both to improve diagnostic yield and to elucidate disease mechanisms that may serve as targets for future therapeutic interventions.

In this study, we describe 21 affected individuals (S1–S21) from 16 unrelated families, all presenting with a distinct form of MD identified through extensive international multicenter screening. Consistent with the recognized clinical heterogeneity of MD, our cohort displayed a broad spectrum of disease severity: most individuals exhibited mild, late-onset symptoms, whereas a subset remained subjectively asymptomatic, in some cases even at advanced age (S3 and S4). Based on fundus imaging, disease presentation could be broadly stratified into three phenotypic types (Type 1–3), which appeared to show some correlation with age. In several cases, irregular and paramacular fundus changes were observed, suggesting that cone involvement may extend beyond the macula, especially in more advanced disease. OCT imaging provided additional structural information and allowed grouping into four morphological types (Type 1–4), which generally corresponded to visual function, with lower OCT grades associated with better visual acuity. Nevertheless, clinical variability remained, with certain asymptomatic individuals showing advanced fundus changes (S6) and others experiencing visual decline despite relatively intact retinal structures on OCT (S21). These observations highlight the complexity and variable expressivity of the condition.

At the molecular level, all affected individuals harbored heterozygous missense variants in *KATNA1* (M1–M10), which encodes the catalytic subunit of the microtubule-severing enzyme katanin. Some variants appeared to be associated with milder disease presentation (for example, M2, which was identified predominantly in asymptomatic individuals who maintained good visual acuity even at advanced age), whereas others, such as M6 (with onset before the age of 30) or M4 (with disease presentation in the early second decade of life in S10), were linked to earlier or more pronounced symptoms. However, overall, no consistent genotype-phenotype correlation could be established across our cohort. Dominant inheritance was documented in five families, whereas most cases were reported as isolated. In one family (Family 9), incomplete penetrance of M5 was demonstrated, as the variant was inherited from the unaffected mother of the proband S13. In other families, parents were not available for clinical and genetic evaluation, and thus, asymptomatic carrier status or *de novo* occurrence could not be excluded. Such phenotypic variability and incomplete penetrance are well recognized features in dominant MDs,^84^ including occult MD (MIM: 613587),^80,85^ Best vitelliform MD (MIM: 153700),^86^ or *PRPH2*-associated pattern dystrophy (MIM: 169150).^87,88^ It is also not uncommon for well-established pathogenic variants in adMD genes to be observed at low frequency in population databases. For instance, one of the most common disease-associated *PRPH2* missense changes (NM_000322.5:c.514C>T, p.Arg172Trp),^89^ has been reported in three European individuals in the latest release of the gnomAD dataset, consistent with its documented variable expressivity.^87^

In total, we identified ten distinct single-nucleotide substitutions affecting six amino acid residues (Thr256, Arg371, Arg372, Arg376, Gly418, and Asp427). Unlike the extensive allelic heterogeneity typically observed across IRD-associated genes,^90,91^ several *KATNA1* variants detected in our study were recurrent across families (M2 in four pedigrees, M6 in three, and M4 in two), suggesting the presence of mutational hotspots, rather than founder alleles, given the diverse ethnic origin of their carriers. This interpretation was further supported by the presence of multiple distinct substitutions affecting the same residues: M1 and M2 both altered Thr256, M4 and M5 affected Arg372, whereas M6, M7, and M8 all changed Arg376. The majority of identified variants clustered within the AAA domain of KATNA1, a highly conserved sequence that contains ATPase activity and is present across all AAA proteins in organisms ranging from protozoa to invertebrates, vertebrates, and higher plants.^49^ As we previously demonstrated,^30^ ATP hydrolysis in the katanin hexamer is activated by arginine residues contributed *in trans* by the adjacent protomer. These amino acids (Arg371 and Arg372) are fully conserved even in unicellular KATNA1 orthologs, and mutations of the corresponding residues in *C. elegans* (Arg351 and Arg352) were shown to inactivate ATPase activity and abolish severing.^30,32^ Since severing requires katanin hexamerization,^92^ mutations that impair oligomerization in *C. elegans* also showed a decrease in this enzymatic activity.^32^ Thus, by analogy and as supported by our structural modeling, all identified KATNA1 substitutions are expected to compromise katanin’s microtubule-severing function.

Consistent with this prediction, patient-derived fibroblasts carrying the recurrent KATNA1 M6 variant exhibited features of reduced microtubule turnover, reflected by increased abundance of hyper-stable microtubules in both ciliary and cytoplasmic compartments. Although KATNA1 expression was preserved in fibroblasts carrying M6, it displayed a distinct subcellular pattern, with retention or mislocalization along polyglutamylated tubulin and ER-associated structures. The ER forms a highly dynamic, interconnected network of tubules and sheets that extends along stable, post-translationally modified microtubules, and its morphology and dynamics are sensitive to changes in microtubule stability.^93,94^ Accordingly, increased abundance of long-lived microtubules resulting from dominant-negative missense mutations in another severing enzyme (*SPAST*) has been linked to ER disorganization and, consequently, lysosomal dysfunction.^93,95^ Consistent with these findings, enlarged lysosomal compartments were also observed in our study, further linking the Arg376 substitution to alterations in cellular homeostasis. While these findings support impaired katanin-associated severing as a plausible disease mechanism, future work to quantify severing activity will be important to define variant-specific effects.

The katanin holoenzyme consists of a catalytic p60 subunit encoded by *KATNA1* and a regulatory p80 subunit encoded by *KATNB1*. While the p80 itself lacks severing activity, it directs p60 to specific subcellular compartments, including the centrosome.^43^ Biallelic variants in *KATNB1* cause a severe syndromic disorder, characterized by microcephaly, lissencephaly, and a range of neurological symptoms, sometimes accompanied by short stature, polysyndactyly, and dental anomalies.^96–98^ Although ocular abnormalities are not reported as part of the *KATNB1*-associated phenotype, additional examinations in two affected siblings noted strabismus, nystagmus, a unilateral macular scar, and “salt and pepper”-like retinal mottling in one, and strabismus with bilateral macular degeneration in the other.^33^ Patient-derived fibroblasts harboring biallelic hypomorphic *KATNB1* variants showed reduced protein levels of both KATNB1 and KATNA1, together with centrosomal abnormalities, while experimental knockdown of *KATNB1* in cultured cells increased microtubule stability,^98^ similar to the results obtained in our study. Consistent with these findings, *Katnb1* was shown to be essential during early embryogenesis in both mice and zebrafish, and its complete absence was embryonically lethal.^98^

Similarly, constitutive knockout of *Katna1* in mice resulted in complete prenatal lethality.^99^ To further evaluate the potential disease mechanism, we considered evidence from population constraint data: among 807,162 individuals included in the latest release of the gnomAD database (v.4.1.0),^38^ no homozygous *KATNA1* loss-of-function (LoF) genotypes were detected. In fact, a total of 86 distinct high-confidence LoF variants (excluding changes located in the last exon) were identified in this repository, but all at the heterozygous state, arguing against haploinsufficiency as a pathogenic mechanism. Instead, the clustering of identified *KATNA1* disease-associated missense variants in conserved functional residues, together with our structural modeling and data obtained from a patient-derived cell line, supports either gain-of-function or dominant-negative effects. The latter appears more likely, given that under random assembly, heterozygous carriers of *KATNA1* variants would theoretically be expected to form only ~1.6% fully wild-type hexamers.

The retina is highly dependent on microtubule organization and function, reflecting its exceptional degree of cellular organization, polarity, and trafficking demands. Microtubules are abundant in multiple retinal layers and support key processes, including vesicle trafficking, ciliary transport, and maintenance of photoreceptor architecture.^100^ In photoreceptors, they are additionally required for the formation of a specialized connecting cilium (CC), which facilitates targeted trafficking and serves as the exclusive conduit between the IS and the OS for all protein and for membrane transport, including rhodopsin and structural components that are crucial for phototransduction.^14^ In addition, the microtubule-based ciliary axoneme extends into the OS, acting as a structural scaffold that provides stability and contributes to disc morphogenesis.^16,101,102^ Accordingly, defects in microtubule regulation, stability, and modifications have previously been implicated in the pathogenesis of both syndromic and non-syndromic IRDs.^103,104^ For example, disease-causing variations in genes encoding axoneme-associated proteins such as *ARL2BP* and *RP1* disrupt microtubule organization or stability within the photoreceptor cilium, leading to progressive retinal degeneration.^102,105,106^ Moreover, an imbalance of microtubule post-translational modifications was shown to affect the photoreceptor CC and destabilize the distal axoneme, promoting photoreceptor degeneration.^27^ Unlike these prior reports, our findings provide the first direct genetic evidence that microtubule severing itself is responsible for photoreceptor health, and thus expand the pathogenic framework of retinal disease to encompass an additional layer of microtubule regulation. To date, three classes of microtubule-severing enzymes have been proposed: katanin, spastin (encoded by *SPAST*), and fidgetin (*FIGN*, MIM: 605295). While the latter has not been associated with human disease, pathogenic variants in *SPAST* are a well-established cause of dominant spastic paraplegia, with a phenotype restricted to neurological features^107,108^ and no retinal abnormalities.^109^ Intriguingly, several *KATNA1* variants identified in the present study map to homologous positions of well-characterized pathogenic *SPAST* variants, suggesting a common molecular dysfunction between these proteins that selectively affects different tissues.

According to RNA-seq data, *KATNA1* shows high retinal expression compared to other human tissues. Immunofluorescence analysis of human retinal sections revealed KATNA1 immunoreactivity restricted to photoreceptors, but with distinct rod and cone labeling patterns. In rods, KATNA1 localized along the axoneme and extended into the distal OS, whereas in cones it was confined mostly to the CC and proximal OS, as also confirmed by TEM results. Subtle cone subtype-specific differences were also observed: diffuse labeling was present in all S cones, whereas a strong signal at the CC was detected in a subset of L/M cones. This latter pattern may reflect either true biological differences between L and M cones or dynamic regulation of *KATNA1* expression depending on cell state or microtubule-severing activity at the time of fixation. The cone CC is a critical bottleneck for protein and membrane trafficking, accommodating the high biosynthetic and metabolic demands of this photoreceptor type through specialized ultrastructural adaptations.^110–112^ Therefore, the selective enrichment of KATNA1 at this site supports a role in regulating microtubule remodeling or stabilization to accommodate the intense trafficking demands of the cone CC.

Surprisingly, we could not detect KATNA1 in the human RPE, as well as in mature iPSC-derived RPE cells. Its staining signal was only observed at week one of iPSC-RPE differentiation, localizing to the retained primary cilia. This could reflect either technical limitations of the detection method or a specific role of KATNA1 in RPE development. Interestingly, the tagged version of *KATNIP* (previously known as *KIAA0556*), encoding katanin-interacting protein (MIM: 616650), was found to localize to the basal body, axoneme, and ciliary tip in hTERT-RPE1 cells.^113^ Variants in this protein are associated with an autosomal recessive ciliopathy, Joubert syndrome 26 (MIM: 616784), in which cone dystrophy has been reported in one family.^114^ Functional studies showed that patient-derived fibroblasts with homozygous *KATNIP* LoF variants had defective ciliogenesis and elongated cilia.^113^

Beyond the retina, *KATNA1* shows high expression in testis, thymus, and skeletal muscles. In the testis, this likely reflects its role in the assembly of microtubule-based structures such as the sperm flagellum, as evidenced by conditional depletion of *Katna1* in male germ cells in mice, which results in defective spermatogenesis and male infertility.^115^ Additionally, in neurons, katanin has been implicated in neurite outgrowth and dendritic remodeling.^44^ While no fertility issues or consistent extra-ocular manifestations were reported in our cohort, the broader role of katanin raises the possibility that subtle additional symptoms could emerge with long-term follow-up.

Taken together, our findings establish heterozygous missense variants in *KATNA1* as a previously unrecognized molecular cause of a non-syndromic macular dystrophy, accounting for approximately 4.1% of MD index cases without prior genetic diagnosis who underwent molecular screening in this study. By integrating genetic, structural, cellular, and expression data, we demonstrate that disrupted microtubule-severing dynamics represent the central pathogenic mechanism and define katanin function as a key regulator of macular homeostasis.

## Methods

### Families and DNA samples

This study adhered to the tenets of the Declaration of Helsinki and was approved by the Ethics Committees of the respective Institutions (Cantonal Committee of Canton Vaud for Research Activities on Human Subjects, Ethikkommission Nordwest- und Zentralschweiz, Ethics Board of the Medical Faculty of the University Tübingen, Comité de Ética del Instituto de Investigación Sanitaria La Fe, London - Camden & Kings Cross Research Ethics Committee, Tel Aviv Sourasky Medical Center, Ethics Committee at Schneider Children’s Medical Center of Israel, Rabin Medical Center, Research Ethics Board, Hospital for Sick Children, CE-AVEN Institutional Review Board). Written informed consent was obtained from all individuals or their legal guardians prior to their inclusion in this study. All study subjects underwent standard ophthalmological evaluation that included best-corrected visual acuity (BCVA), refractive error, multimodal imaging including fundus autofluorescence imaging (FAF), optical coherence tomography (OCT), and a subset of individuals had also full-field electroretinography (ERG) performed.

Genomic DNA was obtained from peripheral blood or saliva samples.

### DNA sequencing and data analysis

Whole-exome sequencing (WES), whole-genome sequencing (WGS), targeted Sanger sequencing, and subsequent data analysis were performed according to protocols specific to each of the participating institutions, but followed a common framework described in detail previously.^8–10,89^

WGS for patients recruited in the United Kingdom was performed as part of the NIHR BioResource Rare Diseases research study (for S6),^116^ UK Genomics England 100,000 genomes project (for S12), and NHS Genomic Medicine Service (for S9, S10, S15, and S16), and bioinformatic analysis was initially carried out using the Genomics England clinical pipeline,^117,118^ with variant filtering restricted to protein-altering variants within the R32 PanelApp Retinal Disorders gene panel.^119^

All identified variants were validated using VariantValidator^120^ and described in accordance with the Human Genome Variation Society (HGVS) nomenclature.^121^ Population allele frequencies were retrieved from large-scale reference datasets, including gnomAD (versions 2.1.1 and 4.1.0),^38^ The All of Us Research Program (version 8),^39^ and GenomeAsia 100k.^40^

Validation of Next-Generation Sequencing data and intrafamilial segregation analysis were performed, where possible, by Sanger sequencing on PCR products, according to standard protocols. Sequences of primers are available upon request.

### Variant interpretation

Identified *KATNA1* variants were classified using the criteria of the American College of Medical Genetics and Genomics (ACMG) and ClinGen Variant Classification Guidance.^41^ Specifically, for the criterion *PP3*, the strength of evidence was assigned according to the REVEL score thresholds.^122^ *PM2_supporting* was applied only to variants that were not present in any of the queried large-scale population databases. *PP1* was assigned for cases with documented intrafamilial segregation, with its strength according to current recommendations.^123^ For variants observed in more than one family, *PS4_strong* was applied based on case-control enrichment calculated using PS4-LRCalc with an odds ratio ≥ 5 and a 95% lower confidence interval.^124^ For this, the aggregated number of cases without prior genetic diagnosis, screened across all participating research centers, was considered the ‘case group’ (comprising 265 individuals with MD and 985 additional IRD cases where the precise phenotype was not specified), and compared against the ‘control group’ represented in gnomAD v4.1.0. Finally, *PM5_moderate* was assigned to variants M1 and M5, as they affect the same amino acid residues as M2 and M4, respectively, which were classified as pathogenic, whereas *PM5_supporting* was assigned to M7 and M8, as both affect the same amino acid residue as M6, classified as likely pathogenic

### Structural analysis

The previously published atomic model of *C. elegans* katanin hexamer in complex with substrate (PDB ID: 6UGD)^32^ was used to examine the structural basis of identified patient-derived variants in atomic details. Sequence alignment between human and *C. elegans* katanin protein was performed, and differences in residue identity and numbering were taken into account when describing the predicted effect of each mutation.^32^ Structural figures were generated with UCSF ChimeraX.^125^

Protein sequence alignments across species, as well as homology analyses between KATNA1 and SPAST, were performed with T-Coffee^126^ and Expresso^127^. MetaDome was employed to detect homologous pathogenic variants between KATNA1 and SPAST,^128^ which were subsequently cross-validated using the ClinVar database.^129^ A schematic plot of variant distribution was created using ProteinPaint.^130^. Protein domain annotations were curated based on UniProt (ID: O75449)^131^ and relevant literature.^32,49^ The mutational constraint landscape across the full KATNA1 sequence was visualized using MutScore.^35^

### Fibroblast cell culture and immunostaining

Primary skin fibroblasts were obtained from a skin biopsy, immortalized by exogenous expression of human telomerase reverse transcriptase (hTERT),^132^ and maintained in DMEM(1x) with GlutaMAX (Gibco), supplemented with 10% fetal bovine serum (FBS, Gibco) and 1% MycoZap (Lonza Bioscience), at 37°C in 5% CO_2_. For immunostaining experiments, cells were seeded on glass coverslips in 24-well plates and allowed to adhere for 24 h. To induce ciliogenesis, cultures were then serum-deprived for 24 h by replacing the growth medium with DMEM (1×) containing GlutaMAX only. Cells were then briefly washed with pre-warmed 1× phosphate-buffered saline (PBS, Merck) and fixed for 15 min with 4% paraformaldehyde (PFA), followed by an additional 7 min fixation with ice-cold 100% methanol. Residual aldehydes were quenched using 100 mM glycine (Merck), and cells were blocked for 1 h at room temperature (RT) in blocking buffer containing 2% bovine serum albumin, 2% normal goat serum, and 0.1% Triton X-100.

Immunofluorescence staining was performed by incubating coverslips with respective primary antibodies overnight at 4°C: anti-ARL13B (PTG, Cat. # 17711–1-AP, 1:1000 dilution), anti-AcTub (Sigma, Cat. # T 6793, 1:1000 dilution), anti-KATNA1 (PTG, Cat. # 17560–1-AP, 1:200 dilution), anti-GT335 (Adipogen, Cat. # AG-20B-0020-C100, dilution 1:1000), anti-PDI (Abcam, Cat. # ab2792, 1:200 dilution), and anti-LAMP1 (Abcam, Cat. # ab25630, 1:100 dilution). Species-appropriate Alexa Fluor-conjugated secondary antibodies (Invitrogen, Cat. #A-11017 and Cat. #A-21430) were used at a 1:500 dilution. Nuclei were stained with DAPI (Thermo Fisher, Cat. # D1306, 1:500 dilution), and the actin cytoskeleton was visualized using Phalloidin-iFluor 647 conjugate (Abcam, Cat. # ab176759, 1:1000 dilution). Coverslips were mounted using ProLong Glass Antifade Mountant (ThermoFisher). Images were acquired using an Olympus scanning confocal microscope equipped with 40x and 63x oil objectives.

For quantitative ciliary analyses, images were collected from three independent technical replicates for each condition. Cilia length measurement and cell count per image were performed using ACDC software,^133^ with manual verification. For acetylated tubulin, total background-corrected fluorescence intensity per image was measured using Fiji,^134^ then normalized to the number of cells, and reported as arbitrary fluorescence units (AU). GraphPad Prism version 10.0.2 was used for statistical analyses and graphical representation of data. Comparisons between groups were assessed using an unpaired Student’s t-test with Welch’s correction. A p-value ≤ 0.05 was considered statistically significant.

### Immunostaining of the human retina

Human retinal paraffin-embedded (FFPE) sections were deparaffinized in xylene and rehydrated through a graded ethanol series. Antigen retrieval was performed in citrate buffer (0.01M, pH 6.0) at 98°C for 15 min. After cooling, sections were permeabilized and blocked for 1 h in 5% normal goat or normal donkey serum. Immunofluorescence staining was then performed with the following primary antibodies overnight: anti-KATNA1 (as above, 1:500 dilution), anti-Tubulin (ABCD Antibodies, Cat. # AA345/AA344, dilution 1:100), and anti-Ezrin (Invitrogen Cat # 35-7300, dilution 1:250). The secondary antibodies used were Alexa Fluor 488 F(ab’)2 goat anti-rabbit IgG (Invitrogen, Cat. # A-11070, 1:500 dilution), Alexa Fluor 647 F(ab’)2 goat anti-mouse IgG (Invitrogen, Cat. # a21237, 1:500 dilution), and CF-658 goat anti-guinea pig IgG (Sigma Aldrich, Cat. # SAB4600080, 1:500 dilution). For cone opsin-specific staining, the primary antibodies included anti-OPN1MW/LW (Santa Cruz, Cat. # sc-14358, 1:10 dilution) and anti-OPN1SW (Santa Cruz, Cat. # sc-14363, 1:10 dilution), while the secondary antibodies used were Alexa Fluor 488 donkey anti-goat (Invitrogen, Cat. # A-11055, 1:500 dilution), Alexa Fluor 647 F(ab’)2 donkey anti-mouse IgG (Jackson ImmunoResearch, Cat. # 715-606-150, 1:500 dilution), and Alexa Fluor 647 F(ab’)2 donkey anti-rabbit IgG (Jackson ImmunoResearch, Cat. # 711-565-152, 1:500 dilution). DAPI (as above) was used for nuclear staining. Wheat Germ Agglutinin, WGA (Vector Labs, Cat. # RL-1022-10, dilution 1:1000), was used to visualize rods, while Lectin Peanut Agglutinin, PNA (Invitrogen, Cat# L32458, dilution 1:1000), labeled cones. The specificity of the staining was confirmed by incubation with secondary antibodies only. Tissues were mounted on slides with ProLong Gold Antifade Mountant (ThermoFisher) and #1.5 glass coverslips. Imaging was performed with an Olympus scanning confocal microscope, using a 63x oil objective. For higher resolution, imaging was performed on a Leica STELLARIS 8 Falcon point scanning confocal microscope, using an HC PL APO 63x/1.40 objective. Lightning processing was performed immediately post-imaging on LASX software.

### Immunostaining of iPSC-RPE cells

Human induced pluripotent stem cell-derived RPE (iPSC-RPE) cells were differentiated from the female iPS(IMR90)-4-DL-01 line, as previously described.^135^ Thereafter, they were seeded on MG-coated trans-wells and cultured as a monolayer for 1, 3, and 5 weeks, respectively. At these timepoints, cells were fixed using 4% PFA in PBS for 15 min. For immunofluorescence staining, membrane inserts were removed from trans-wells, sectioned, and stained as described previously.^70^ Immunofluorescence staining was performed with the following primary antibodies: anti-KATNA1 (as above, 1:500 dilution) and anti-GT335 (as above). Secondary antibodies used were: Alexa Fluor 488 F(ab′)2 goat anti-mouse IgG, Alexa Fluor 594 F(ab′)2 goat anti-rabbit IgG; DAPI was used for nuclear staining, and Phalloidin-iFluor 647 conjugate labeled actin filaments (all as described above). The iPSC-RPE sections were then mounted on slides using VectaShield Antifade Mounting Media (Vector Labs) and covered with #1.5 glass coverslips. Imaging was performed with an Olympus scanning confocal microscope, using a 63× oil objective.

### Immunogold transmission electron microscopy

Following human retina dissection, the retinas were pre-fixed with 4% PFA in Ames’ media (Sigma) for 15 min and prepared as previously described.^136^ Immunolabelling was performed with anti-KATNA1 (as above, 5ug) as a primary antibody and Nanogold-Fab’ goat anti-rabbit (Nanoprobes Cat# 2004, 15ul) as a secondary antibody. Retinas were post-fixed for 1 h at RT in 2.5% PFA, 2.5% glutaraldehyde, 4 mM CaCl_2_ in 0.3 M cacodylate buffer, pH 7.4. Aldehydes were quenched in 100 mM glycine (Merck) prepared in PBS. Silver enhancement was performed using HQ Silver Kit (Nanoprobes, Yaphank, NY). En bloc staining with 1% tannic acid, 0.5% glutaraldehyde in 0.1 M HEPES (pH 7.5), and 1% uranyl acetate in 0.1 M maleate buffer (pH 6.0) followed, before ethanol dehydration and embedding in Eponate resin. 70 nm ultramicrotome sections were cut from the resin blocks using a Diatome Ultra 45° diamond knife and collected onto copper slot-grids (VWR). Grids were post-stained in Uranyless (EMS Hatfield, Pennsylvania, USA) for 10 min and lead citrate solution (EMS) for 10 min. Grids were imaged on a FEI Tecnai Spirit (FEI Company) transmission electron microscope, operated at 120 keV using a side-mounted 2K × 2K CCD camera (Veleta, Olympus). Radius software was used for image acquisition, and images were subsequently cropped with slight contrast adjustments in Fiji.^134^

## Supplementary Files

This is a list of supplementary files associated with this preprint. Click to download.

- TableS2v3.xlsx
- TableS1v6.xlsx
- SupplementaryFigures.pdf
- nrreportingsummaryKATNA12.pdf

**Figure S1. Bilateral multimodal retinal imaging of individuals with *KATNA1*-associated macular dystrophy.** Images from fourteen individuals with available complete multimodal datasets are shown. Each panel represents one subject, with the corresponding family (FX), subject ID (SX), age at examination (yo - years old), and identified *KATNA1* variant (MX). In each panel, the top row shows color or pseudocolor fundus images, the middle row displays fundus autofluorescence (FAF), and the bottom row shows macular spectral-domain optical coherence tomography (OCT) scans. Clinical classification based on FAF and OCT for each eye is indicated on the respective image (T - type). OD - right eye, OS - left eye.

**Figure S2. Conservation and homology of the identified *KATNA1* variants. A.** Sequence alignment of parts of KATNA1 with orthologues from vertebrates, invertebrates, plants, and unicellular organisms. Variants detected in this study (M1–M10) are shown in red, and the corresponding conserved residues across species are highlighted in bold. All missense changes affect residues that are either fully or highly conserved across all organisms. **B.** Mutational constraint across the full KATNA1 protein, shown as a plot of running MutScore average per ten amino acids (green line; range 0–1, where 1 indicates the highest predicted deleteriousness). Domain organization of KATNA1 is depicted below with color-coded features. Protein positions of the variants identified in this study are marked by red lines. **C.** Alignment of the AAA core domain (Pfam ID: PF00004) sequences of KATNA1 and SPAST. KATNA1 variants identified in this study within this domain (M1–M8) are shown in red, and homologous residues in SPAST with reported pathogenic mutations are marked in bold.

**Figure S3. Expression of *KATNA1* in human tissues.** Expression values across selected human tissues and organs from the Human Protein Atlas (bulk RNAseq), based on the GTEx (**A**) and FANTOM5 (**B**) datasets. Only tissues with KATNA1 expression ≥10 nTPM (normalized transcripts per million, A) or ≥20 scaled tags per million (B) are shown.

**Figure S4. Immunofluorescence detection of KATNA1 in human retina and iPSC-RPE cells. A.** Schematic representation of the photoreceptor and RPE layers of the retina. Human retina sections with preserved RPE were stained with KATNA1 (green) together with WGA (magenta, left panels), or tubulin (magenta), and Ezrin (grey) (right panels). KATNA1 immunoreactivity was detected in the outer segments (OS) of photoreceptors, with no specific signal observed in the RPE layer. WGA highlights rod OS, while Ezrin labels the microvilli of the RPE. Nuclei were counterstained with DAPI (blue). Scale bars are indicated in the respective panels. Abbreviations: RPE - retinal pigment epithelium; OS - photoreceptor outer segments, IS - inner segments; ONL - outer nuclear layer. **B**. Monolayers of iPSC-derived RPE cells, analyzed at 1, 3, and 5 weeks of maturation. Staining included KATNA1 (green), GT335 (magenta, labeling glutamylated tubulin), phalloidin (orange, marking the actin cytoskeleton), and DAPI (blue). At week one, arrows highlight retained primary cilia co-stained with GT335 and KATNA1, consistent with early RPE maturation. At later stages (3 and 5 weeks), cells acquired mature epithelial organization, characterized by prominent actin outlining hexagonal cell borders, and no specific KATNA1 signal was observed. Scale bars are indicated in the respective panels.

## Figures and Tables

**Table 1.** General, clinical, and genetic information of the affected individuals included in this study.

General information					Clinical information				Genotype	
Family ID	Subject Study ID	Sex	Family relationship	Age at examination (years)	Main diagnosis	Age at onset (years)	Ocular symptoms	Concomitant conditions	Variant (ID)	*KATNA1* (NM_007044.4)
**Family 1**	**S1**	M	proband	62	MD	61	reduced VA, light sensitivity	arterial hypertension	**M1**	c.766A>G, p.(Thr256Ala)
**Family 2**	**S2**	M	proband	25	MD	curr. asympt.	none	none	**M2**	c.767C>T, p.(Thr256Met)
	**S3**	F	mother	62	MD	curr. asympt.	none	none	**M2**	c.767C>T, p.(Thr256Met)
	**S4**	M	uncle	67	MD	curr. asympt.	none	none	**M2**	c.767C>T, p.(Thr256Met)
**Family 3**	**S5**	F	proband	35	MD	unknown	night vision difficulty	none	**M2**	c.767C>T, p.(Thr256Met)
**Family 4**	**S6**	M	proband	29	MD	curr. asympt.	none	arterial hypertension	**M2**	c.767C>T, p.(Thr256Met)
**Family 5**	**S7**	M	proband	36	MD	unknown	night vision difficulty	none	**M2**	c.767C>T, p.(Thr256Met)
**Family 6**	**S8**	F	proband	38	MD	37	reduced VA, metamorphopsia	none	**M3**	c.1112G>A, p.(Arg371Gln)
**Family 7**	**S9**	F	proband	7	MD	curr. asympt.	none	infantile eczema, pityriasis alba	**M4**	c.1114C>T, p.(Arg372Cys)
	**S10**	F	sister	11	MD	10	difficulty reading	none	**M4**	c.1114C>T, p.(Arg372Cys)
	**S11**	M	father	44	MD	unknown	reduced VA	none	**M4**	c.1114C>T, p.(Arg372Cys)
**Family 8**	**S12**	M	proband	49	MD	48	metamorphopsia	none	**M4**	c.1114C>T, p.(Arg372Cys)
**Family 9**	**S13**	M	proband	6	MD	curr. asympt.	none	none	**M5**	c. 1115G>A, p.(Arg372His)
**Family 10**	**S14**	F	proband	62	MD	18	reduced VA, night vision difficulty	overweight	**M6**	c.1127G>A, p.(Arg376Gln)
**Family 11**	**S15**	F	proband	9	MD	curr. asympt.	none	none	**M6**	c.1127G>A, p.(Arg376Gln)
**Family 12**	**S16**	F	proband	38	MD	27	metamorphopsia	vitamin D deficiency, underwent thyroidectomy	**M6**	c.1127G>A, p.(Arg376Gln)
**Family 13**	**S17**	F	proband	27	MD	curr. asympt.	none	none	**M7**	c.1127G>C, p.(Arg376Pro)
**Family 14**	**S18**	F	proband	57	MD	20	reduced VA, night vision difficulty	pancreatic cysts	**M8**	c.1127G>T, p.(Arg376Leu)
**Family 15**	**S19**	M	proband	55	MD	35	metamorphopsia, mild night vision difficulty	mild bilateral hearing loss, asthma, vertigo, osteoporosis	**M9**	c.1253G>A, p.(Gly418Asp)
	**S20**	M	brother	58	MD	42	metamorphopsia	mild bilateral hearing loss, hydronephrosis, mitral valve prolapse	**M9**	c.1253G>A, p.(Gly418Asp)
**Family 16**	**S21**	M	proband	60	MD	58	reduced VA	none	**M10**	c.1279G>C, p.(Asp427His)

F - female, M - male; MD - macular dystrophy; curr. asympt. - currently asymptomatic; VA - visual acuity

## Data Availability

All variants identified in this study will be submitted to the ClinVar database (https://www.ncbi.nlm.nih.gov/clinvar/). The authors declare that all relevant datasets that are necessary to interpret and verify the research in this article are included within the manuscript and its supplementary information files, or are available upon request. Data from the National Genomic Research Library (NGRL) used in this research are available within the secure Genomics England Research Environment. Access to NGRL data is restricted to adhere to consent requirements and protect participant privacy. Data used in this research include whole-genome sequencing data (located in the /genomes/by_date folder) and associated phenotypic information extracted using Participant Explorer, from participants enrolled in the Genomics England Rare Disease Programme. Access to NGRL data is provided to approved researchers who are members of the Genomics England Research Network, subject to institutional access agreements and research project approval under participant-led governance. For more information on data access, visit: https://www.genomicsengland.co.uk/research.
